# Changes in macular sensitivity after half-dose photodynamic therapy for chronic central serous chorioretinopathy

**DOI:** 10.1186/s12886-017-0535-y

**Published:** 2017-08-10

**Authors:** Alp Atik, Yijun Hu, Honghua Yu, Chun Yang, Bin Cai, Yijing Tao, Dongli Li, Yan Chen, Li Lu, Guodong Li, Ling Yuan

**Affiliations:** 1 Department of Ophthalmology, The First Affiliated Hospital of Kunming Medical University, 295 Xichang Road, Kunming, 650031 China; 2 Royal Victorian Eye and Ear Hospital, Melbourne, Australia; 3 Shanwei Project Vision Eye Hospital, Shanwei, China; 4 Department of Ophthalmology, General Hospital of Guangzhou Military Command of PLA, Guangzhou, China; 5 Department of Ophthalmology, The People’s Hospital of Gejiu City, Gejiu, China; 6 Department of Ophthalmology, The Second Affiliated Hospital of Nanchang University, Nanchang, China

**Keywords:** Central serous chorioretinopathy (CSCR), Photodynamic therapy (PDT), Visual field, Macular sensitivity, Automated static perimetry

## Abstract

**Background:**

To evaluate the macular sensitivity changes after half-dose photodynamic therapy (PDT) for chronic central serous chorioretinopathy (CSCR).

**Methods:**

Eighteen patients (26 eyes) with chronic CSCR were recruited in the same hospital between April 2011 and December 2012. All patients were treated with one session of half-dose PDT after complete ophthalmic examination. Macular sensitivity examination was performed at baseline and 1, 3 and 6 months post-treatment. Mean sensitivity (MS) of the central 10 degrees (10°) and 4 degrees (4°), mean deviation (MD) and pattern standard deviation (PSD) on automated static perimetry (Humphrey Field Analyzer II-750) were used for analysis.

**Results:**

There was significant improvement of the 10°MS from baseline (29.76 ± 1.51 dB) to 1 month (31.74 ± 1.56 dB), 3 months (31.51 ± 1.38 dB) and 6 months (31.19 ± 1.61 dB) after treatment (*P* < 0.001). The 4°MS was also significantly improved with half-dose PDT from baseline (28.96 ± 1.78 dB) to 1 month (32.41 ± 1.66 dB), 3 months (32.46 ± 1.50 dB) and 6 months (31.90 ± 1.84 dB) post-treatment (*P* < 0.001). MD was improved from baseline (−3.39 ± 0.89 dB) to 1 month (−1.96 ± 0.29 dB), 3 months (−1.94 ± 0.29 dB) and 6 months (−2.45 ± 0.13) post-treatment (*P* = 0.004). PSD also improved from 1.97 ± 0.24 dB at baseline to 1.47 ± 0.27 dB, 1.34 ± 0.24 dB, and 1.53 ± 0.24 dB (*P* = 0.001) at 1, 3 and 6 months after treatment, respectively.

**Conclusion:**

Macular sensitivity in CSCR can be improved by half-dose PDT, along with improvement of visual acuity and retinal thickness. The treatment outcome at 1 month may be a predictor of the final treatment response.

## Background

Central serous chorioretinopathy (CSCR) is characterized by serous retinal detachment and sometimes retinal pigment epithelial (RPE) detachment, most often confined to the macula and associated with leakage of fluid through the RPE into the subretinal space [1]. CSCR can occur in acute or chronic forms, with 3 months as the most widelyused differentiating parameter [1]. The acute form often presents with a moderate reduction in best-corrected visual acuity (BCVA) and generally resolves spontaneously with minimal sequelae. However, recurrence is quite common, occurring in 30–50% of patients within 1 year [2]. In contrast, chronic CSCR can result in widespread retinal damage, with photoreceptor death, chronic neurosensory retinal detachment, RPE atrophy, choroidal neovascularization (CNV) and permanent loss of vision [3–5].

Indications for treatment of CSCR include persistent or chronic CSCR, recurrence in eyes with visual deficits from CSCR, visual deficits in the other eye from CSCR, presence of chronic changes and occupational reasons. Photodynamic therapy (PDT) with verteporfinhas been widely used for the treatment of CSCR with promising results [6–11]. PDT has an occlusive effect on CNV and also causes choroidal hypoperfusion [12]. It is also purported to decrease choroidal hyperpermeability and tighten the blood retinal barrier at the RPE, countering subretinal fluid accumulation [13, 14]. However, these effects can also cause complications such as RPE atrophy, juxtafoveal CNV and transient reduction in macular function [15–19]. Thus, ‘safety-enhanced’ (half-dose verteporfin) PDT is now more commonly used in the treatment of CSCR [6, 7, 20, 10].

Macular sensitivity is usually reduced in CSCR [21, 22], especially in the chronic form [21]. Studies have shown that half-dose PDT can improve macular sensitivity along with improvement of BCVA [23–26]. Most of these studies used microperimetry to assess macular sensitivity [24–26]. However, microperimetry is not usually available in many hospitals. In contrast, automated static perimetry (ASP) is more commonly used in many settings. Studies have shown that the results of ASP is significantly correlated with those of microperimetry [27, 28]. Hence it seems reasonable to assess macular sensitivity using ASP when microperimetry is not available. In the present study, we used ASP to evaluate macular sensitivity in patients with chronic CSCR after half-dose verteporfin PDT treatment.

## Methods

### Study participants

Eighteen patients (26 eyes) with chronic CSCR were recruited in the same hospital between April 2011 and December 2012. All study subjects were male. Informed consent was obtained from each patient. This prospective study was in agreement with the Declaration of Helsinki and approved by the Institutional Review Board of the First Affiliated Hospital of Kunming Medical University.

Inclusion criteria included: 1. BCVA 20/200 or better, 2. visual symptoms associated with CSCR (such as decrease vision, central scotoma) lasting for more than 3 months, 3. presence of subretinal fluid involving the fovea on optical coherence tomography (OCT), 4. active characteristic angiographic leakage (a focal leakage point in the early phase with pooling of fluorescein in the later phases) on fluorescein angiography (FA), 5. presence of choroidal hyperpermeability on indocyanine green angiography (ICGA) [11].

Exclusion criteria were acute CSCR with disease duration less than 3 months, previous PDT or focal laser photocoagulation for CSCR, evidence of other macular diseases (such as age-related macular degeneration, polypoidal choroidal vasculopathy, diabetic macular edema, retinal vein occlusion), corneal opacity, glaucoma or ocular hypertension, refractive error greater than ±6.00 diopters and a history of ocular surgery, intraocular inflammation, ocular trauma and optic neuropathy.

All patients underwent a complete ophthalmic examination, including BCVA with decimal charts, slit-lamp examination and indirect ophthalmoscopy. Macular sensitivity examination was performed with the Humphrey Field Analyzer II-750 (Carl Zeiss Meditec, Dublin, CA, USA) using SITA-FAS strategy of the 10–2 program at baseline and at each follow-up (1, 3 and 6 months) after treatment. The stimulus size was Goldmann III and the background luminance was 31.5ASB. Visual fields with fixation loss <15%, false positive <20%, false negative <20% were considered as reliable. Mean sensitivity (MS) of the central 10 degrees (10°) and 4 degrees (4°), mean deviation (MD) and pattern standard deviation (PSD) were used for analysis. The MD and PSD were automatically calculated by the Humphrey Field Analyzer II-750. The 10°MS was calculated as the mean of the 68 point sensitivity within the central 10° area and the 4°MS was calculated as the mean of the 16 point sensitivity within the central 4° area.

Central foveal thickness (CFT) was measured at baseline and at each post-treatment follow-up with OCT (STRATUS OCT 3000, Carl Zeiss Meditec, Dublin, CA). FA and ICGA (Heidelberg Retina Angiograph II, Heidelberg Engineering, Heidelberg, Germany) were also performed simultaneously at baseline and at 6 months follow-up.

Patients were treated with one session of half-dose verteporfin PDT after confirmation of diagnosis. Verteporfin was administered intravenously at a dose of 3 mg/m^2^ body surface area within 8 min, followed by PDT at 10 min from the commencement of infusion to cover the area of leakage on FFA and choroidal hyperpermeability on ICGA using the following parameters: wavelength 689 nm, light intensity 600 mW/cm^2^, duration 83 s, dose 50 J/cm^2^.

### Statistical analysis

The main outcome measures BCVA, CFT, 10°MS, 4°MS, MD and PSD were assessed by normality test. The main outcome measures between baseline and different post-treatment follow-ups were compared with one-way ANOVA. Two-tailed Student’s *t*-test was used for the comparison of two time-points. Pearson linear correlation was used to assess the correlation between any two of the main outcome measures. *P* < 0.05 was considered statistically significant.

## Results

### Basic characteristics

Twenty-six eyes of 18 patients with CSCR were included in the study. The mean age of the patients was 43.2 years (range: 32–48 years). All patients were male. Eight patients had CSCR in both eyes and 10 patients had CSCR in one eye. Most of the patients had relatively well preserved RPE.

### BCVA

There was significant improvement in BCVA from baseline (0.44 ± 0.22) to 1 month (0.69 ± 0.19), 3 months (0.73 ± 0.18) and 6 months (0.70 ± 0.18) post-treatment (ANOVA F = 11.535,*P* < 0.001). All the post-treatment time-points were significantly different to baseline (all *P* < 0.001). There was no significant difference in BCVA between any of the post-treatment time-points (*P* > 0.05) (Tables 1 and 2).

**Table 1:** Main outcome measures at baseline and different follow-ups (mean ± SD)

Variable	baseline	1 month	3 months	6 months	F	p
BCVA	0.44 ± 0.22	0.69 ± 0.19	0.73 ± 0.18	0.70 ± 0.18	11.535	<0.001
CFT	404.84 ± 28.34	261.38 ± 26.69	227.38 ± 25.57	233.53 ± 23.69	27.227	<0.001
MD	−3.39 ± 0.89	−1.96 ± 0.29	−1.94 ± 0.29	−2.45 ± 0.13	4.744	0.004
10°MS	29.76 ± 1.51	31.74 ± 1.56	31.51 ± 1.38	31.19 ± 1.61	7.108	<0.001
4°MS	28.96 ± 1.78	32.41 ± 1.66	32.46 ± 1.50	31.90 ± 1.84	17.830	<0.001
PSD	1.97 ± 0.24	1.47 ± 0.27	1.34 ± 0.24	1.53 ± 0.24	6.329	0.001

**Table 2:** *p* value of comparison of main outcome measures

Variable	baseline vs 1 month	baseline vs 3 months	baseline vs 6 months	1 month vs 3 months	1 month vs 6 months	3 months vs 6 months
BCVA	<0.001	<0.001	<0.001	0.483	0.888	0.574
CFT	<0.001	<0.001	<0.001	0.136	0.221	0.786
MD	0.002	0.001	0.036	0.957	0.270	0.248
10°MS	<0.001	<0.001	0.003	0.629	0.244	0.494
4°MS	<0.001	<0.001	<0.001	0.918	0.372	0.319
PSD	0.003	<0.001	<0.001	0.434	0.520	0.890

### CFT

Subretinal fluid resolved completely by 1 month in 23 eyes (88.5%) and by 3 months in 2 eyes. Only one eye had unresolved subretinal fluid at 6 months. CFT was significantly reduced from baseline (404.84 ± 28.34um) to 1 month (261.38 ± 26.69um), 3 months (227.38 ± 25.57um) and 6 months (233.53 ± 23.69um) post-treatment (F = 27.227, *P* < 0.001). Mean CFT from all the post-treatment time-points was significantly lower than that at baseline (all *P* < 0.001). Although there was a further decrease in the mean CFT from 1 month to 3 and 6 months post-treatment, the difference between the time-points was not significant (all *P* > 0.05) (Tables 1 and 2).

### FFA and ICGA

All of the eyes had leakage within the foveal avascular zone (FAZ) on FFA. FFA showed that leakage stopped in 24 eyes (92.3%) at 6 months after half-dose PDT. There was reduced leakage in 1 eye (3.84%) and no change in 1 eye (3.84%). No patient developed complications such as RPE atrophy and secondary choroidal ischemia as seen on ICGA after treatment.

### Macular sensitivity

There was significant improvement in the 10°MS from baseline (29.76 ± 1.51 dB) to 1 month (31.74 ± 1.56 dB), 3 months (31.51 ± 1.38 dB) and 6 months (31.19 ± 1.61 dB) after treatment (F = 7.108, *P* < 0.001). All three time-points were significantly different to baseline (*P* < 0.001, <0.001, =0.003 at 1, 3 and 6 months respectively). There was no significant difference between any two post-treatment time-points (all *P* > 0.05) (Tables 1 and 2).

The 4°MS was also significantly improved with half-dose PDT from baseline (28.96 ± 1.78 dB) to 1 month (32.41 ± 1.66 dB), 3 months (32.46 ± 1.50 dB) and 6 months (31.90 ± 1.84 dB) post-treatment (F = 17.830, *P* < 0.001). All three time-points (1 month, 3 months and 6 months) were significantly different to baseline (all *P* < 0.001). Comparison of 4°MS between any two postoperative follow-ups was not significant (all *P* > 0.05) (Tables 1 and 2). Moreover, the 4°MS was lower at baseline but was more significantly improved after treatment than the 10°MS, suggesting that the foveal function was more compromised by the disease but was more restored after treatment than the entire macula.

The half-dose PDT treatment significantly improved the MD from baseline (−3.39 ± 0.89 dB) to 1 month (−1.96 ± 0.29 dB), 3 months (−1.94 ± 0.29 dB) and 6 months (−2.45 ± 0.13) post-treatment (F = 4.744, *P* = 0.004). All three time points (1 month, 3 months and 6 months) were significantly improved compared with baseline (*P* = 0.002, 0.001, 0.036 respectively). There was a decrease in mean MD from 3 months to 6 months post-treatment but this was not significant (*P* > 0.05) (Tables 1 and 2).

Half-dose PDT also improved the PSD (F = 6.329, *P* = 0.001). The baseline PSD improved from 1.97 ± 0.24 dB to 1.47 ± 0.27 dB (*P* = 0.003), 1.34 ± 0.24 dB (*P* < 0.001), and 1.53 ± 0.24 dB (*P* < 0.001) at 1, 3 and 6 months after treatment, respectively. Despite a slight rise at 6 months, the difference in the PSD between any two post-treatment follow-ups was not significant (all *P* > 0.05) (Tables 1 and 2).

### Correlation of main outcomes

There was modest negative correlation between CFT and BCVA (*r* = −0.295, *P* = 0.002), CFT and 10°MS (*r* = −0.314, *P* < 0.001) and CFT and MD (*r* = −0.198, *P* = 0.038). This meant that with the resolution of subretinal fluid, the macular functions (BCVA, 10°MS and MD) were also improved.

## Discussion

CSCR is generally considered to be a self-limited condition. However, some patients can experience significant visual impairment caused by recurrent attacks of CSCR, persistent chronic neurosensory retinal detachment, or RPE atrophy. Since many CSCR patients are working age, this visual impairment (especially if chronic) may adversely interfere with daily activities and productivity, thus enhancing the requirement for safe, effective treatment.

Our results demonstrate that macular sensitivity as assessed with ASP can be improved after half-dose verteporfin PDT. This is clinically important as visual acuity often does not capture the visual disturbances of CSCR patients. Half-dose PDT resulted in significant improvement in BCVA and macular sensitivity along with resolution of subretinal fluid after treatment. In addition, FFA showed a cessation of leakage in 92.3% of patients at 6 months after treatment. None of the patients in our study experienced visual loss (or other complications) after PDT with half-dose verteporfin. No patient developed complications shown to be associated with full-dose verteporfin PDT, such as secondary CNV, RPE atrophy and secondary choroidal ischemia.

Several studies have evaluated the effects of half-dose PDT on macular sensitivity in patients with chronic CSCR using microperimetry. The results were promising and the macular sensitivity improved in most patients up to 12 months after treatment [24–26]. However, microperimetry is not commonly available in many hospitals. On the contrary, ASP is commonly used in general practice. Our results demonstrate that ASP can also be used to assess macular sensitivity in CSCR patients. Springer et al. showed that the results of ASP are comparable with microperimetry in healthy volunteers [27]. Inpatients with macular diseases whose visual acuity is low and fixation is poor, ASP may not perform as well as microperimetry [29]. However, patients with CSCR usually have good visual acuity and fixation even when the disease is chronic [21, 24]. To ensure good central fixation during macular sensitivity examination using ASP, we ruled out patients whose BCVA was worse than 20/200. Our results demonstrating improved macular sensitivity as assessed by ASP were consistent with those of other studies using microperimetry [24–26].

We feel that the improvements in MS, MD and PSD seen in our study are both statistically and clinically significant. In a previous study Squirrell et al. has demonstrate that a change of >1.5 dB in MS detected by microperimetry can be regarded as a significant change in visual function in wet AMD patients [30]. The mean change of 10°MS in our study was 1.98 dB at 1 month, 1.75 dB at 3 months and 1.43 dB at 6 months. The mean change of 4°MS in our study was 3.45 dB at 1 month, 3.5 dB at 3 months and 2.94 dB at 6 months. Most of the MS changes in our study were more than 1.5 dB. In a study by Frennesson et al. the mean improvement of MD in wet AMD patients successfully treated with ranibizumab was at least 27% from the baseline MD [31]. In our study, the mean improvement of MD from baseline was 42.2% at 1 month, 42.8% at 3 months and 27.7% at 6 months. All of the improvement was more than 27%. Moreover, a less extent of change in MS and MD would be expected in CSCR patients. This is because patients with better central vision usually have smaller variation in MS and MD [32] and CSCR patients usually have better central vision than wet AMD patients.

In our study, BCVA was significantly improved at 1 month after treatment and remained stable at 3 months and 6 months. Consistently, 10°MS and 4°MS were also improved in the same manner, suggesting that macular function as well as foveal function improved after treatment. This is because in a majority of CSCR cases the subretinal fluid is not limited to the subfoveal area, usually extending to the juxtafoveal region and in some cases even beyond, to involve the whole macula. In chronic CSCR the photoreceptors at the areas of subretinal fluid may undergo dysfunction and degeneration and causes visual disturbance. Some photoreceptors may partially regain function with resolution of the subretinal fluid, which is reflected by the increase in 10°MS and 4°MS. However, our results indicate that macular function is not fully restored after treatment, with MD at post-treatment follow-ups higher than baseline, but still lower than normal subjects. An interesting finding of our study is that the foveal function recovery seem to be more significant than the entire macula. This is consistent with some previous studies. Sekine et al. have shown a lower MS at the fovea compared to that at the outer macular area in CSCR [33]. Ehrlich et al. have also shown that improvement of 6°MS is more profound than 12°MS at 3 months and 6 months after PDT treatment for CSCR [34].

Sanguansaket al. found that macular sensitivity in eyes with resolved CSCR after half-dose PDT was still lower than the normal fellow eyes [23]. The loss and recovery of photoreceptor function seems to be more significant at the fovea, as suggested by the results of our study. Lower BCVA before treatment was associated with higher PSD, indicating that foveal function was more damaged compared to other regions of the macula. Similarly, higher BCVA after treatment was associated with lower PSD, suggesting that recovery of foveal function was more significant than other macular areas. Our findings are consistent with Piccolino et al. who found that photoreceptor damage in CSCR is more profound at the fovea [35].

Interestingly, we showed no significant difference in treatment response with any of the macular sensitivity parameters between 1, 3 and 6 months. Most of the patients who showed improvement in macular sensitivity at 1 month post-treatment did not demonstrate a further response to half-dose PDT at 3 or 6 months. Clinically, this suggests that success of half-dose PDT treatment may be determined early, with the potential to utilize other treatment modalities after assessing response at 1 month.

Our study had several limitations, including small sample size. We did not obtain data on choroidal thickness, as can be done by enhanced depth imaging OCT. It would be interesting to assess whether baseline choroidal thickness is significantly altered by half-dose PDT and whether this correlates with treatment response. We were also only able to report 6 months follow-up results and further research is required to assess patients at 1 year or longer. In addition, although severe damage to the retina or choroid did not occur within the trial period, further follow-up is needed to establish long-term safety. Our results are not generalizable to all CSCR patients given the small and short term nature of this study, but they do indicate that automated static perimetry may play a useful role in evaluating treatment response in this patient population.

## Conclusion

In conclusion, macular sensitivity may be improved by half-dose PDT along with improvement of BCVA and CFT in chronic CSCR. The treatment outcome at 1 month may be a predictor of the final treatment response. Our results showing improvement in macular sensitivity in the chronic CSCR patients treated with half-dose PDT provide further evidence supporting the role of half-dose PDT treatment in this patient population.

## Abbreviations

ASP: Automated static perimetry
BCVA: Best-corrected visual acuity
CFT: Central foveal thickness
CNV: Choroidal neovascularization
CSCR: Central serous chorioretinopathy
FA: Fluorescein angiography
ICGA: Indocyanine green angiography
MD: Mean deviation
MS: Mean sensitivity
OCT: Optical coherence tomography
PDT: Photodynamic therapy
PSD: Pattern standard deviation
RPE: retinal pigment epithelial
